# Knowing Too Much

**Published:** 1887-12

**Authors:** 


### KNOWING TOO MUCH.

It was Alexander Pope who said, "A little knowledge is a dangerous thing," but it was reserved for a later generation to discover that there is such a thing as knowing too much.

Once in a while it happens that a great statesman or soldier is taken ill with some obstinate and grievous disorder. His family physician is called, and appreciating the gravity of the case, and the eminence of his patient, he quickly decides to call in help. Several distinguished specialists are summoned, the case accurately diagnosticated and treatment instituted.

The newspapers contain daily reports of the case, together with descriptions of the wonderful instruments and methods used in the diagnosis and treatment. The public marvels at the great advance of medical science, and shekels fall thick into the coffers of the distinguished specialists as a consequence of the free advertising the case gives them.

Sooner or later it is discovered that the healing art cannot save the patient; that although the specialists are very brilliant in diagnosis, they are a little "short" in therapeutics, or else it will happen that even the diagnosis was wrong, and in spite of a favorable prognosis, the good man is gathered unto his fathers. Then the public laughs at the alleged advances which medical science has made.

Such instances do more to bring contempt upon the profession of medicine than all the fatal mistakes of half-educated doctors. In other words, there is such a thing as knowing too much. This was exemplified several years ago in the case of President Garfield; it is also being illustrated at the present time by the illness of the Crown Prince of Germany.

In Garfield's case, some of the most distinguished surgeons in the land were in attendance. They succeeded in making themselves absurd in the eyes of the profession and the public; their consultations were characterized by wavering indecision, and nothing was done to save the great man's life. It is true a brilliant genius proposed to locate the position of the bullet by means of a telephonic probe, and succeeded in placing it in the right groin, but the autopsy showed it was about eighteen inches from this spot.

Several months ago, Dr. Morell MacKenzie, the great English throat specialist, removed a growth from the larynx of the Crown Prince of Germany. He gave it as his opinion that the growth was perfectly benign, and there was not the least danger of a recurrence. In order to "make assurance doubly sure," a specimen of the growth was sent to Professor Virchow, whose reputation as a pathologist is world-wide. After examining it, he declared without hesitation that it was simply "pachyderma verrucosa," and was in no way malignant.

Such an announcement had the effect of entirely allaying the fears of the German people, for their faith in Virchow is unbounded.

Unfortunately, the growth *has* recurred in the patient's larynx, and there is no longer any doubt of its cancerous nature.

Such a terrible blunder on the part of two such distinguished men as MacKenzie and Virchow cannot fail to bring the science of medicine into ridicule and contempt.

The whole trouble was that they knew too much; great confidence had robbed them of caution, and they left themselves no loop-hole for escape in case they should be mistaken. It is very seldom that the *average* doctor is caught committing himself so positively on a question of diagnosis and prognosis, but with an ingenuity born of his very ignorance, he hedges his opinions, so that in any event the friends will say, "It is just as the doctor said."

Dr. MacKenzie and Professor Virchow could learn a lesson from their less-learned brethren.

In the homely, but forcible language of Josh Billings, "It is better not to know so much than to know so many things that ain't true."

